# Case Report: Concomitant Alzheimer's and Lewy-Related Pathology Extending the Spectrum of Underlying Pathologies of Corticobasal Syndrome

**DOI:** 10.3389/fnins.2021.742042

**Published:** 2021-11-03

**Authors:** Michaela Kaiserová, Katerina Menšíková, Lucie Tučková, Petr Hluštík, Petr Kaňovský

**Affiliations:** ^1^Department of Neurology, University Hospital, Palacky University, Olomouc, Czechia; ^2^Department of Neurology, Faculty of Medicine and Dentistry, Palacky University, Olomouc, Czechia; ^3^Department of Clinical and Molecular Pathology, University Hospital, Palacky University, Olomouc, Czechia

**Keywords:** corticobasal syndrome, Alzheimer's related pathology, Lewy-related pathology, corticobasal degeneration, concomitant pathologies

## Abstract

Corticobasal syndrome (CBS) is clinically characterized by progressive asymmetric rigidity and apraxia together with symptoms suggestive of cortical involvement and basal ganglia dysfunction. The spectrum of neurodegenerative diseases that can manifest with CBS is wide. The associations of CBS with corticobasal degeneration, progressive supranuclear palsy, Alzheimer's disease, frontotemporal lobar degenerations, Creutzfeldt–Jakob disease, or diffuse Lewy body pathology have been reported. We describe the case of a 71-year-old woman with CBS. The histopathological examination of brain tissue revealed concomitant pathology corresponding to the limbic stage of Lewy-related pathology and the intermediate stage of Alzheimer's-type pathology. To date, there have been only a few cases with a similar combination of pathology manifesting with the CBS phenotype that have been described in the literature. The extent and distribution of pathological changes in these cases were somewhat different from ours, and significance for clinical manifestation was attributed to only one of these pathologies. In our case, we assume that both types of pathology contributed to the development of the disease, considering the presumed specific spread of both types of pathological processes according to Braak's staging. Our case expands the spectrum of neurodegenerative pathological processes that may manifest with the typical CBS phenotype. Also, it points out the importance of identifying specific biomarkers that would enable more accurate *in vivo* differential diagnosis and more accurate determination of the underlying pathological processes of these diseases.

## Introduction

Corticobasal syndrome (CBS) is clinically characterized by progressive asymmetric rigidity and apraxia together with symptoms suggestive of cortical involvement (e.g., alien limb phenomena, cortical sensory loss, myoclonus, or mirror movements) and basal ganglia dysfunction, e.g., bradykinesia, dystonia, or tremor (Boeve, 2011). The term CBS was introduced for this combination of symptoms, once considered a unique clinical manifestation of corticobasal degeneration (CBD), a disease of significant clinico-pathological heterogeneity (Casseron et al., 2005). The spectrum of neurodegenerative diseases that can manifest with CBS is wide: CBS can be associated with CBD, progressive supranuclear palsy (PSP), Alzheimer's disease (AD), frontotemporal lobar degeneration with ubiquitin and TDP-43 positive inclusions (FTLD-TDP), frontotemporal lobar degeneration with fused in sarcoma-positive inclusions (FTLD-FUS) or Creutzfeldt–Jakob disease (CJD) (Riley et al., 1990; Rinne et al., 1994; Tsuboi et al., 2005; Vandenberghe et al., 2007; Imamura et al., 2009; Ling et al., 2010; Tartaglia et al., 2010; Saranza et al., 2019). Association with diffuse Lewy body pathology is also reported (Horoupian and Wasserstein, 1999; Kasanuki et al., 2018; Nishida et al., 2019).

Here, we present a patient with a pathological finding corresponding to a concomitant limbic type of Lewy-related pathology (or Braak stage V of Lewy body disease) (McKeith et al., 1996; Braak et al., 2003) and intermediate category of Alzheimer's-type pathology (A1, B3, C3) (Mirra et al., 1991; Montine et al., 2012), who presented with the clinical phenotype of CBS as another example of the clinicopathological heterogeneity of the CBS/CBD spectrum.

## Case Description

### Clinical Data

A 71-year-old, right-handed Caucasian female of Czech origin was examined and followed up in the tertiary Movement Disorders Center. She was referred with a 2-year history of progressive memory loss and speech difficulties. Her initial problems further included difficulties with activities of daily living and decreased ability to make decisions, clumsiness of the right hand with loss of manual dexterity on performing fine motor tasks, and an olfactory disorder, which lasted ~2 years. On admission, neurological examination revealed bradykinesia, gait disturbance, right-side rigidity, motor aphasia, ideomotor apraxia, and right-side hemiparesis. In more detail, the increased tendon reflex responses were present aside the pathological exteroceptive reflexes including Juster's sign, Trömner's sign, and upper forearm sign. The plastic muscle tone is increased together with postural reflexes, and cog-wheel phenomenon was present. There was no velocity- and length-dependent responses present. Based on a combination of clinical symptoms, the clinical diagnosis of “probable” CBS was made (Armstrong et al., 2013).

Magnetic resonance imaging (MRI) of the brain showed chronic post-ischemic changes in the white matter of both hemispheres and cerebral atrophy of the temporal lobes with a predominant involvement of the left hemisphere. In the cerebrospinal fluid (CSF), there was an increase in total tau protein (t-tau) level and increased t-tau/amyloid-beta (Aβ)_42_ ratio.

A detailed speech examination classified the disorder as severe integration aphasia with impaired visual perception and apraxia. Neuropsychological examination revealed severe dementia, a score of 15/30 was obtained on the Mini-Mental State Examination (MMSE); memory impairment was also noted, particularly in the domains of implicit memory and recognition. There was also a disorder of numerical skills, a deficit of verbal fluency in the lexical and semantic component, and severe dysexecutive syndrome. Examination of the brain by perfusion single-photon enhanced computed tomography (SPECT) showed a markedly asymmetric involvement with a severe alteration of perfusion in the parietal lobe on the left side, extending into the left temporal and occipital lobes.

**Figure 1:**
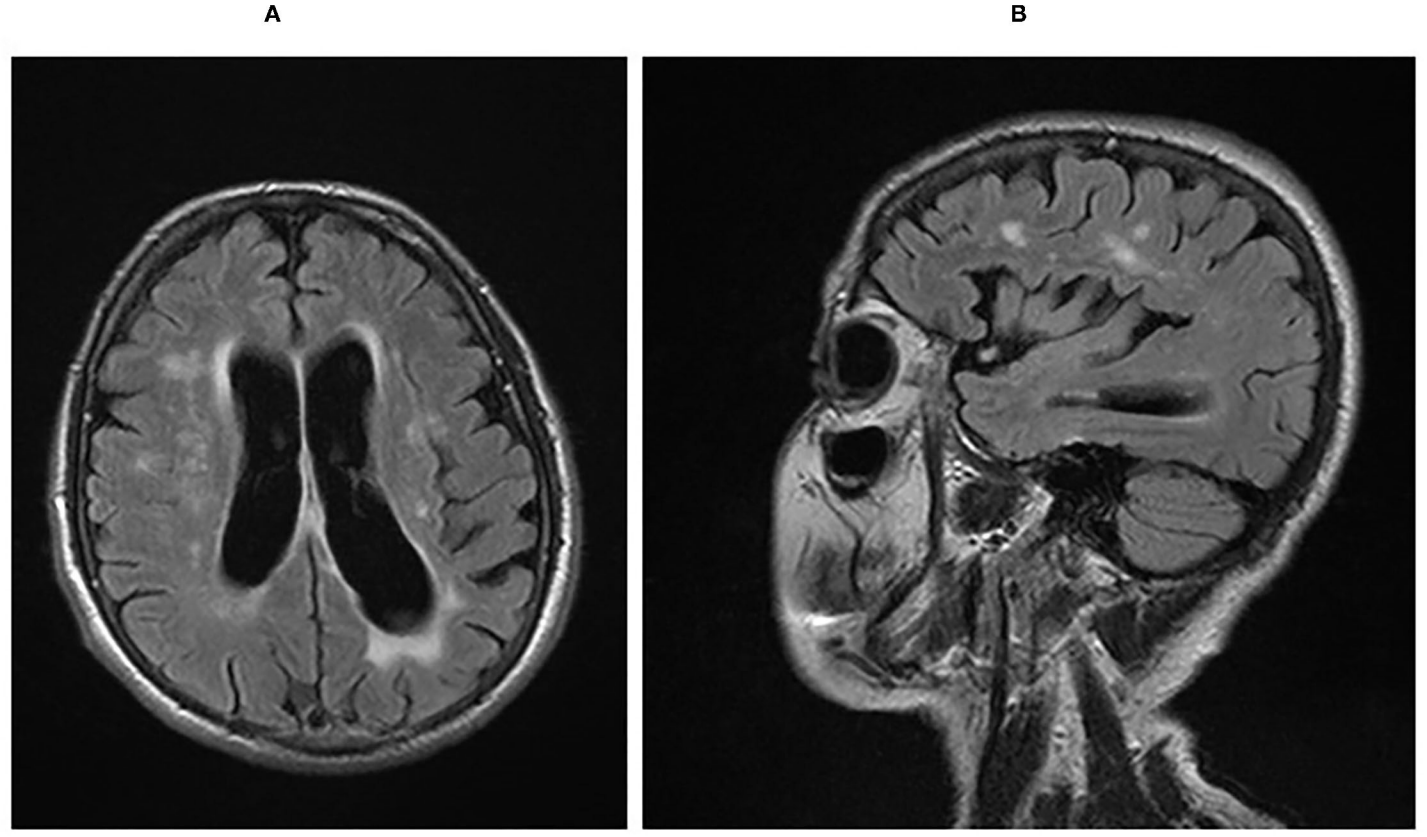
**(A)** Sagittal FLAIR MRI sequence showing cerebral atrophy with a frontotemporal predilection and post-ischemic hyperinsities in the white matter. **(B)** Axial FLAIR MRI sequence showing asymmetry of frontotemporal atrophy with left-side predominance. FLAIR, fluid-attenuated inversion recovery; MRI, magnetic resonance imaging.

As the disease progressed, apraxia worsened, and the alien hand sign developed on the right side. Repeated speech examination 3 years after the disease onset revealed significant deterioration in all tested domains. Progression of Parkinsonian signs was noted: limb rigidity (more pronounced on the right side), bradykinesia, shuffling gait, and reduced arm swings; eye movement apraxia and myoclonic jerks were also recorded. MRI of the brain at this stage showed brain atrophy with frontotemporal predominance (Figure 1A), again with more severe impairment on the left side (Figure 1B). Five years after disease onset, the patient was bedridden, suffering from severe dementia, aggression, visual hallucinations, and occasional epileptic seizures. In addition, severe muscular hypertonus with a combination of rigidity and spasticity and with dystonic posture of the limbs was present and more prominent on the right side. The patient died at the age of 78 years after 7 years of disease course. A detailed neuropathological examination of brain tissue was performed after the consent of the next of kin was obtained.

### Neuropathology

**Figure 2:**
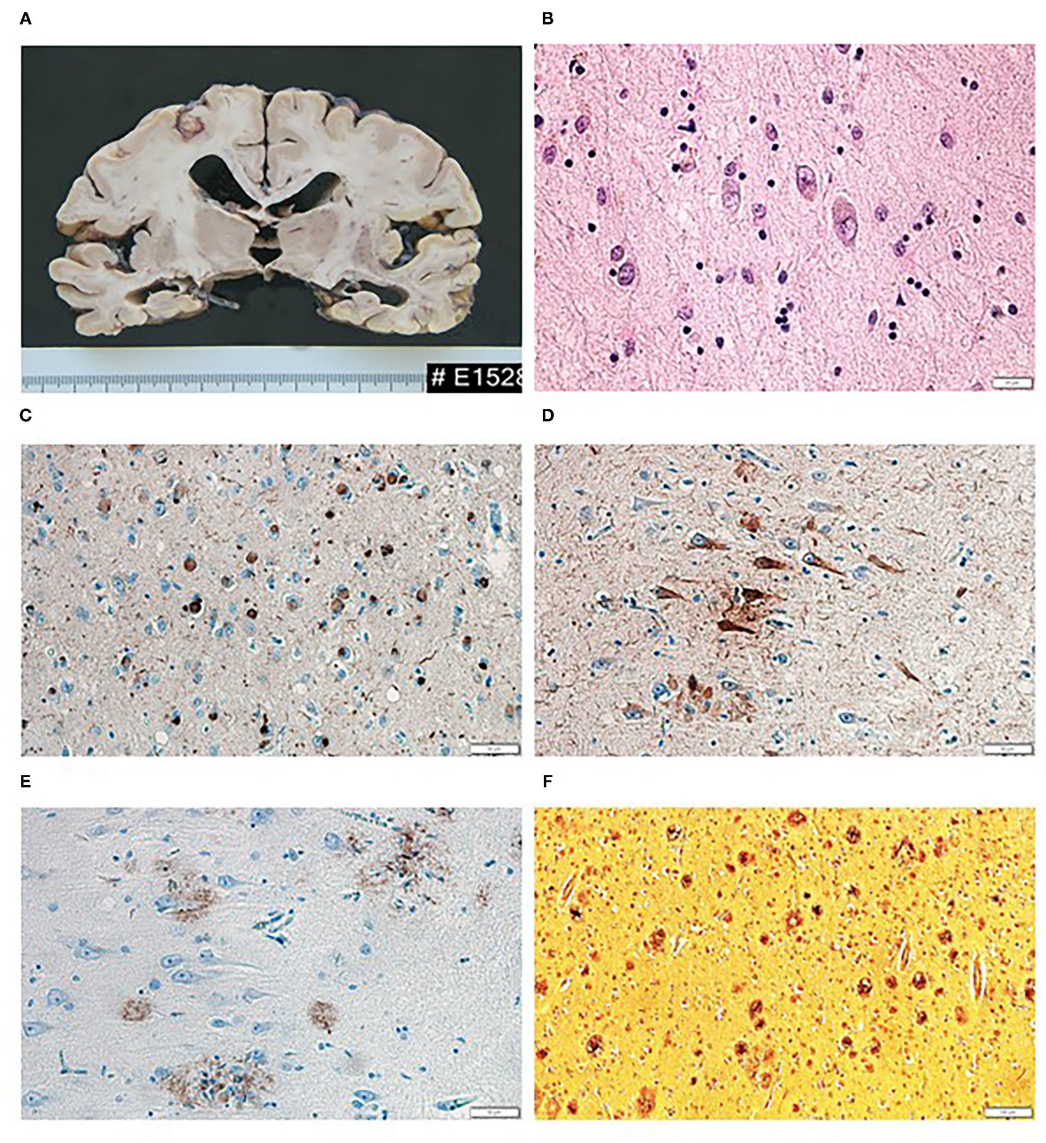
Macroscopic and microscopic findings of histopathological examination of brain tissue. **(A)** Diffuse brain atrophy with the compensatory dilatation of the lateral and third ventricles; the atrophy was most pronounced in the frontotemporal regions, particularly in medial temporal lobes. **(B)** Lewy bodies in the neurons of the amygdala (HandE, magnification 400×). **(C)** Lewy bodies, dystrophic Lewy neurites and dots in the amygdala (α-syn 5G4, magnification 200×). **(D)** Neurofibrillary tangles and threads in the hippocampus (AT8, magnification 200×). **(E)** β-amyloid deposits in the hippocampus (β-amyloid, magnification 200×). **(F)** Plaques in the frontal cortex (AgNOR, magnification 100×).

At the autopsy, the brain macroscopically showed diffuse symmetrical atrophy (total fixed brain weight was 1,050 g) with compensatory dilatation of the lateral and third ventricles; the atrophy was most pronounced in the frontotemporal regions, particularly in the medial temporal lobes (Figure 2A). The substantia nigra was slightly depigmented, and the locus coeruleus was not visible at all. There were present severe arteriosclerotic changes with extensive calcifications within the vessels of the Willis circle, and the cribrous state of the basal ganglia was also recorded.

Microscopically, 1- to 2-μm-thick sections of formalin-fixed paraffin-embedded tissue from specific regions of the brain were examined: frontal, temporal, parietal, occipital and motor cortices, cingular gyrus, hippocampus and parahippocampal region, basal ganglia, thalamus, midbrain at the level of substantia nigra, pons, oblongata at the level of inferior olivary nucleus, and cerebellum.

In the routine hematoxylin and eosin staining, loss of neurons associated with gliosis was found in the substantia nigra and locus ceruleus together with neuronal cytoplasmic pale bodies and classic Lewy bodies (Figure 2B).

The immunohistochemical examination revealed α-synuclein positivity in the following regions: midbrain, pons, oblongata, basal ganglia, amygdala, hippocampus and insular and temporal cortices; frontal and parietal cortices were negative. In the brainstem, Lewy bodies were identified as well as granular cytoplasmic α-synuclein positivity, thick or thin dystrophic Lewy neurites and dots; similar deposits were also found in the basal ganglia and the adjacent insular cortex where they were numerous. In contrast, there were only sporadic pathological inclusions present within the temporal cortex. Abundant Lewy bodies, dystrophic neurites and dots were found in the amygdala (Figure 2C), and the hippocampus and the cingular gyrus were similarly affected.

Alongside Lewy-type pathology, we also identified typical Alzheimer's-type pathological signs. The immunohistochemistry showed tau-protein positive deposits in the following regions: hippocampus, amygdala and cingular gyrus, neocortical regions (temporal, occipital, frontal, parietal, and motor cortices), basal ganglia, thalamus, and brainstem (midbrain and pons) (Figure 2D). The pathological inclusions were found in the form of pre-tangles, tangles, and numerous threads; in most regions, the density was either moderate or high (in contrast to the brainstem, where it was only sparse). The immunohistochemical examination with β-amyloid antibody revealed extracellular parenchymal diffuse and focal deposits—plaques in the tissue samples taken from the neocortex (frontal, temporal, parietal, and occipital) and hippocampus (Figure 2E); other regions were negative. The silver impregnation (AgNOR) showed numerous diffuse, primitive as well as neuritic plaques in the cortices as well as in the limbic regions (Figure 2F). In the silver impregnation, only the neuritic plaques were evaluated. The results of the microscopic examination with the severity of the pathological finding in individual brain areas are shown in Table 1. In conclusion, the overall pathological picture corresponded to the limbic type of Parkinson's disease (Braak stage V) (McKeith et al., 1996; Braak et al., 2003) and the intermediate category of Alzheimer's-type pathology (A1B3C3) (Mirra et al., 1991; Montine et al., 2012).

**Table 1:** Results of the microscopic assessment.

	**α-Synuclein**	**Tau**	**β-Amyloid**	**Silver**	**Phospho-TDP-43**
Mesencefalon	2+	1+	Negative	N/E	N/E
Pons	2+	1+	Negative	N/E	N/E
Oblongata	2+	N/E	Negative	N/E	N/E
Cerebellum	N/E	0	Negative	N/E	N/E
Frontal cortex	0	3+	Positive	3+	Negative
Gyrus cinguli	1+	3+	N/E	N/E	N/E
Striatum	1+	2+	Negative	N/E	N/E
Insular cortex	3+	2+	Positive	N/E	N/E
Amygdala	3+	3+	N/E	2+	Negative
Hippocampus	2+	3+	Positive	3+	Negative
Temporal cortex	0	3+	Positive	3+	N/E
Motor cortex	N/E	3+	N/E	N/E	N/E
Parietal cortex	N/E	2+	Positive	3+	N/E
Occipital cortex	N/E	2+	Positive	2+	N/E

*Evaluation strategy: α-synuclein- evaluated in accordance to the Braak system; tau- evaluated in accordance to the Braak system; β-amyloid- Positive/Negative; phospho-TDP-43, Positive/Negative; silver impregnation (AgNOR), evaluated in accordance with the CERAD system; N/E, Not examined*.

## Discussion

The phenotype of the presented case met the clinical diagnostic criteria of CBS (Mathew et al., 2012) and also those for CBD (Armstrong et al., 2013). Initial symptoms included progressive memory loss, clumsiness of the right upper limb during fine tasks, integration aphasia, ideomotor apraxia, bradykinesia, right-sided Parkinsonian syndrome, and gait disorders, followed by the development of the alien limb phenomenon, myoclonus and lateralized dystonic posture of the limbs. The overall pathological picture corresponded to the concomitant limbic type of Lewy-related pathology and the intermediate category of Alzheimer's-type pathology.

On retrospective evaluation, there were several symptoms that could indicate possible underlying Lewy-related pathology. These were the olfactory disorder, asymmetric manifestations of parkinsonism and visual hallucinations. Olfactory disorder is also reported in CBS cases (Pardini et al., 2009); in these cases, however, neuropathological examination of the brain tissue was not performed, so it is not known what type of pathology was the basis of this disorder. Parkinsonian symptoms initially responded well to dopaminergic treatment (later, the responsiveness turned out to be only partial, which might be the consequence of concomitant tauopathy). On the other hand, there were no other characteristic Lewy bodies disorder signs, such as REM sleep behavioral disorder or fluctuations in cognitive deficit. Among the symptoms that could indicate the presence of Alzheimer's-type pathology, the patient manifested a progressive memory disorder (from the early onset of the disease). One of the clues could be the early deterioration of episodic memory, which is more specific for AD than for CBD or other dementia types (Day et al., 2017) similarly to a difference in the degree of semantic and episodic memory impairment. It is explained by the different distribution of the underlying pathological process, i.e., the mesial temporal lobe in AD and the frontal lobe in CBD (Pillon et al., 1995; Massman et al., 1996; Kertesz and McMonagle, 2010; Day et al., 2017). Other authors also state that compared with individuals with AD, those with CBD had “relative preservation” of memory early in the disease course (despite subjective memory complaints) but an accelerated rate of decline on measures of story recall and letter fluency (Machado de Oliveira et al., 2017). Recognition of these features could help distinguish patients with cognitive complaints due to CBD from those with complaints attributable to AD (Day et al., 2017). Apraxia, one of the basic and early symptoms of CBS, has been repeatedly linked to parietal lobe dysfunction (Gross and Grossman, 2008). Apraxia usually appears in the late stages of AD (Della Sala et al., 1987) although cases with early constructional or ideomotor praxis disability are also described (Rapcsak et al., 1995; Nielson et al., 1996; Mendez, 2019). A study focusing on the detection of neuroanatomical correlates of apraxia in AD found a statistically significant relationship between the density of neurofibrillary tangles in the anterior cingulate cortex and ideomotor apraxia (Giannakopoulos et al., 1998). Apraxia can, thus, manifest already in the prodromal so-called limbic stage of AD, i.e., stages III and IV according to Braak and Braak (1995). Aphasia is also considered a late symptom of AD, which usually develops in the neocortical stage (Braak stages V and VI). However, early manifestations in the form of PPA are reported, similar to cases in which PPA was the only symptom of AD. The pathological correlate in these cases was the involvement of the left peri-sylvian cortex (Greene et al., 1996; Rogalski et al., 2016). Myoclonus, a rare late symptom of AD (Hauser et al., 1986) was in several cases present in the earlier stages of the disease as a clinical correlate of significant AD-type pathology in the motor cortex (Hauser et al., 1986; Horoupian and Wasserstein, 1999).

An increased level of t-tau in the CSF and an increase in the t-tau/(Aβ)_42_ ratio could be another clue to the assumption of underlying Alzheimer's-type pathology. Although the CSF profile of biomarkers in atypical parkinsonian syndromes accompanied by dementia is not yet clearly known, it is thought that increase of the t-tau/Aβ42 ratio in CSF may help in the differential diagnosis of AD from other types of dementias (Irwin et al., 2013; Přikrylova-Vranova et al., 2014).

The co-occurrence of Lewy-related and Alzheimer's-type pathology manifesting as CBS has so far been described only in a few cases (Horoupian and Wasserstein, 1999; Kasanuki et al., 2018; Nishida et al., 2019). The extent and distribution of pathological changes in these cases were somewhat different from ours. In all the previous cases, there was a diffuse Lewy body pathology that met the criteria for dementia with Lewy bodies. The CBS phenotype is attributed to motor cortex involvement with either severe Lewy body pathology (Kasanuki et al., 2018) or severe AD pathology or a combination of both (Horoupian and Wasserstein, 1999; Kasanuki et al., 2018). In contrast, in our case, the overall pathological picture corresponded to the limbic type of Parkinson's disease (McKeith et al., 1996) and the intermediate category of Alzheimer's-type pathology (Mirra et al., 1991; Montine et al., 2012). Cortical involvement, including the motor cortex, included purely intermediate Alzheimer's-type pathology without the presence of Lewy bodies. The CBS phenotype is also described in association with atypical focal AD with a predominant pathology in the motor cortex (Jagust et al., 1990; Golaz et al., 1992). Therefore, the distribution of pathology, rarely also involving the motor cortex, appears to be a factor that plays a role in the development of the clinical picture in the form of CBS.

Considering the development of clinical symptoms in the presented case along with presumed specific spread of both types of pathological processes according to Braak (Braak and Braak, 1995; Braak et al., 2003), we suppose that both types of pathology were involved in the development of the described disease. The initial olfactory disorder, asymmetric parkinsonism present since the onset of the disease, and early episodic memory impairment along with an increase in t-tau/Aβ_42_ ratio in the CSF could be considered as major features that reflected both underlying pathologies. This case extends the spectrum of neurodegenerative pathological processes that may manifest with typical CBS phenotype. At the same time, it highlights the importance of identifying specific biomarkers that would enable more accurate clinical differential diagnosis and more accurate determination of the underlying pathological processes and potential therapeutic targets.

## Data Availability Statement

The raw data supporting the conclusions of this article will be made available by the authors, without undue reservation.

## Ethics Statement

The studies involving human participants were reviewed and approved by Ethics Committee of the University Hospital Olomouc, Czech Republic. The patient/participant provided her written informed consent to participate in this study.

## Author Contributions

MK and KM: clinical examination and assessment of the patient, long-term follow up, and writing of the first draft of manuscript. LT: pathological examination and writing of the first draft of manuscript. PH and PK: critical reading and revision of the final version of the manuscript. All authors contributed to the article and approved the submitted version.

## Funding

This study was supported by grant projects from the Ministry of Health of the Czech Republic—NV19-04-00090 and NV18-04-00346; grant from the Ministry of Health, Czech Republic for the conceptual development of a research organization (FNOL, 0098892); and by European Regional Development Fund - Project ENOCH (No. CZ.02.1.01/0.0/0.0/16_019/0000868), IGA-LF-2021-020.

## Conflict of Interest

The authors declare that the research was conducted in the absence of any commercial or financial relationships that could be construed as a potential conflict of interest.

## Publisher's Note

All claims expressed in this article are solely those of the authors and do not necessarily represent those of their affiliated organizations, or those of the publisher, the editors and the reviewers. Any product that may be evaluated in this article, or claim that may be made by its manufacturer, is not guaranteed or endorsed by the publisher.
